# The Provision of Private Healthcare Services in European Countries: Recent Data and Lessons for Universal Health Coverage in Other Settings

**DOI:** 10.3389/fpubh.2021.636750

**Published:** 2021-03-15

**Authors:** Dominic Montagu

**Affiliations:** ^1^Department of Epidemiology and Biostatistics, University of California, San Francisco, San Francisco, CA, United States; ^2^Metrics for Management, Baltimore, MD, United States

**Keywords:** private sector, Universal Health Coverage, health policy, health system governance, health seeking behavior

## Abstract

Universal Health Coverage (UHC) exists in all of the countries of Europe, despite variation on the ownership structure of health delivery systems. As countries around the world seek to advance UHC and manage the private sector within their health systems, the European experiences can offer useful insights. We found four different models for the provision of healthcare, with the private sector predominant in some countries, and of minimal importance in others. The European experiences indicate that UHC can be effectively provided with, or without, large-scale private sector provision in hospital, specialty, and primary care services, and that moreover it can be provided with high levels of patient satisfaction. These findings offer regulatory models for countries in other regions to review as they advance UHC.

## Background

There is a global movement to make healthcare accessible for those in need, assuring Universal Health Coverage in all countries by 2030. While pursuing this, many Low- and Middle-Income Countries (LMICs) continue to struggle with how and how much to integrate private providers into the formal government regulated and funded health system. This is a very immediate question in countries, such as Nigeria, India, and Myanmar where well over 50% of all services provided are private and quality assurance is a challenge, but also relevant to countries, such as Ethiopia or Vietnam where private care is below 25% and policy makers must wonder if higher levels would accelerate investments in coverage and care availability (1–3).

Ministerial level platforms like the Joint Learning Network use case studies to provide examples for health officials on key policies related to financing and governance which can advance Universal Health Coverage (4, 5). Case studies on health reforms have been used to demonstrate important lessons on regulatory changes and the system and health outcomes that result (6). Researchers hope to understand how the divisions in public-private service ownership affect critical health system indicators, such as efficiency, morbidity, mortality, and equity. This descriptive paper seeks to establish a categorization of systems and provide a foundational first step for future research in both OECD and LMIC settings. Healthcare services in Europe are effective, appreciated by their citizens, and delivered with many different models and degrees of private involvement (7, 8). In the push for UHC, Europe can provide insights into differing experiences with private provision in the context of nationally managed systems. This study provides an up-to-date review of private provision across different sectors in countries across Europe. The experiences are relevant to many settings.

### Financing Context

Provision of healthcare functions independently of financing and there is more competition, more variance, and more change within the ownership, incentives, and regulation of care provision than is the case with financing. Nevertheless, financing sets the context for ownership, together with policy and regulatory guidance, directly or indirectly determining what ownership mix can develop.

**Figure 1:**
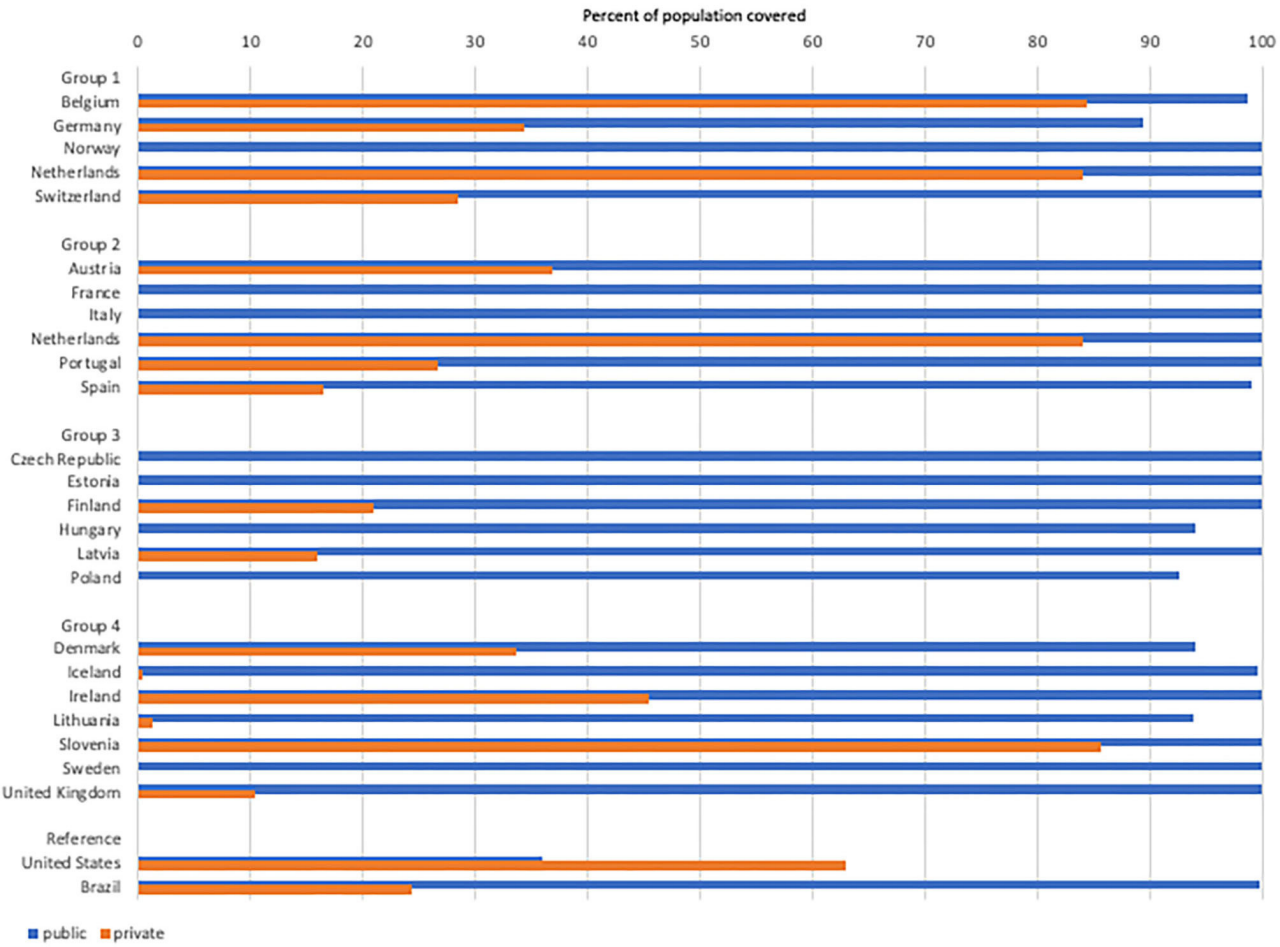
Public and private insurance coverage. Blue bars show population covered by public insurance. Orange bars show population with private insurance*. *Private insurance is supplemental to public in all countries except the US, where private coverage is often a substitute for public insurance. Countries showing zero private insurance coverage did not report any to the OECD in 2018. (Source: https://stats.oecd.org/).

Universal Health Coverage (UHC) exists in all of the European countries we studied. Unlike LMICs, healthcare financing in Europe is almost universally government managed, either directly through taxation revenue (as in the UK) or semi-directly through mandated, managed, and government subsidized Social Health Insurance (as in Germany). Across Europe, government and social health insurance provide a healthcare safety net for nearly all citizens as shown by data from the OECD health system survey (Figure 1, blue bars). While the form of insurance varies between countries, and supplemental private insurance (orange bars) is common in some (Belgium, Holland, Slovenia) but not others (France, Norway), the most important implication for service provision, is that where they exist, private providers in most countries are paid either by national health insurance systems or by tightly regulated social health insurance schemes that coordinate purchasing (4–6). Out of pocket payments for healthcare are consistently low across all European countries surveyed, totaling <0.5% of spending on preventative care and <20% of Total Health Expenditure in 2018 (9, 10). The lesson for other countries is that government purchasing and regulation are neither a guarantee of, nor a barrier to a large private market for healthcare provision.

## Methods

### Scope and Focus

We restricted our analysis to European countries which are members of the OECD. We excluded EU members which were not also OECD members, and OECD countries outside of Europe. Turkey is an OECD country and partially on the European continent, however 97% of the landmass is in Asia and we made a decision to exclude it from this analysis for that reason. In this paper, for the sake of simplicity, we refer to the selected countries as “Europe.”

### Data Sources

We reviewed all publications on the included countries' health systems from the OECD and WHO European websites. For each country we also searched for journal publications in English through PubMed and Google Scholar, and where data was contradictory or lacking we conducted subject specific Google Scholar searches by country (e.g., “dentist Luxembourg”) for additional sources from white papers. Where all of these sources failed, we contacted experts within WHO and personal connections within academic institutions in the countries with information gaps for supplemental sources in other languages.

When calculating the scale of the private sector role within each country we relied heavily on the Health System in Transition (HSiT) national reports from the European Observatory on Health Systems and Policies. These ranged in date-produced from 2003 (Iceland) to 2019 (Latvia) (11, 12). If country-specific reports use pre-2008 data, regardless of when they were published, we set them aside, and instead used data from the 2008/9 OECD health system survey (8). When journal publications or national reports had credible national data which was more recent than either the 2008/9 Survey or the national HSiT report, we used that source. The year of data used for each country is shown in a Supplementary Material.

We applied the healthcare service categories used by the OECD to look separately at inpatient services, specialist services, primary care, and pharmacies (8, 13). We use hospitals as a proxy for inpatient services, this reflecting the majority of providers and care delivered in hospitals across all countries surveyed (14). Outpatient Specialist services and dentistry are treated together. Primary Care could be either general practitioners (UK) or primary care centers (Sweden). And pharmacies here refer only to community pharmacies and so exclude hospital-based pharmacies.

### Patient and Public Involvement

This study used publicly available data to look at health-systems behaviors. No patients were involved, and no direct data collection was undertaken which would have prompted public involvement.

## Results and Discussion

**Figure 2:**
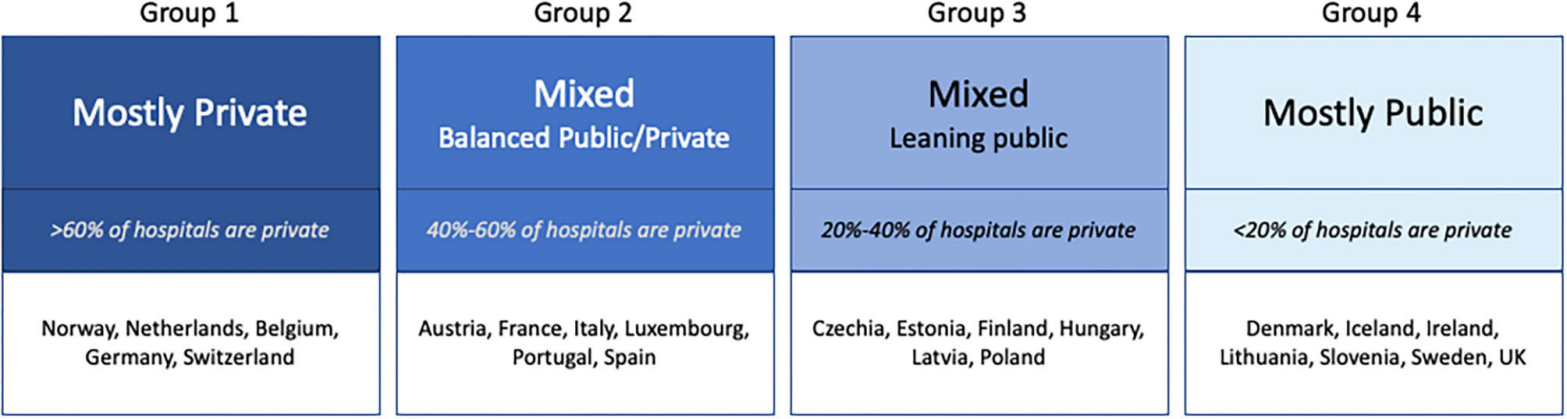
Hospital ownership models within European health systems. Data not available for: Greece and Slovakia.

We evaluated each country on hospital ownership data and then reviewed for consistency against other aspects of care provision. From this we grouped the health systems in Europe into four types (Figure 2), based on how reliant the overall system is on private provision. This grouping was informed by analyses of the interaction between regulatory and purchasing agencies of government and privately owned providers of care across health service domains (15–17). Health systems are highly path-dependent (18, 19) and the four types, or Groups, reflect the continued influence of the financing and ownership models which created current structures. In Germany, the influence of the Bismarkian model of social insurance and privately contracted delivery remains evident (20). In the UK, the influence of Beveridge's vision for the National Health System continues to resonate in current days (21). Nevertheless, as Kutzin argued convincingly a decade ago already, the distinctions between European health systems are becoming less important as financing models align, driven by aging populations and growing expectations for care so that government funding fills more and more gaps in traditional social health insurance, while competition is increasingly common in national health insurance systems to manage costs (22–24).

### Hospitals

Hospitals are in transition across Europe as outpatient services shift outside of medical facilities and most countries push for increased efficiency as measured by shorter average stays and higher bed usage rates (14, 15). Our findings from countries with more recent data showed little change from the ownership status summarized in a 2008/2009 survey among OECD countries (8). Across all European countries the role, and importance, of private hospitals within the larger health system fall into four distinct categories (Figure 2).

**Figure 3:**
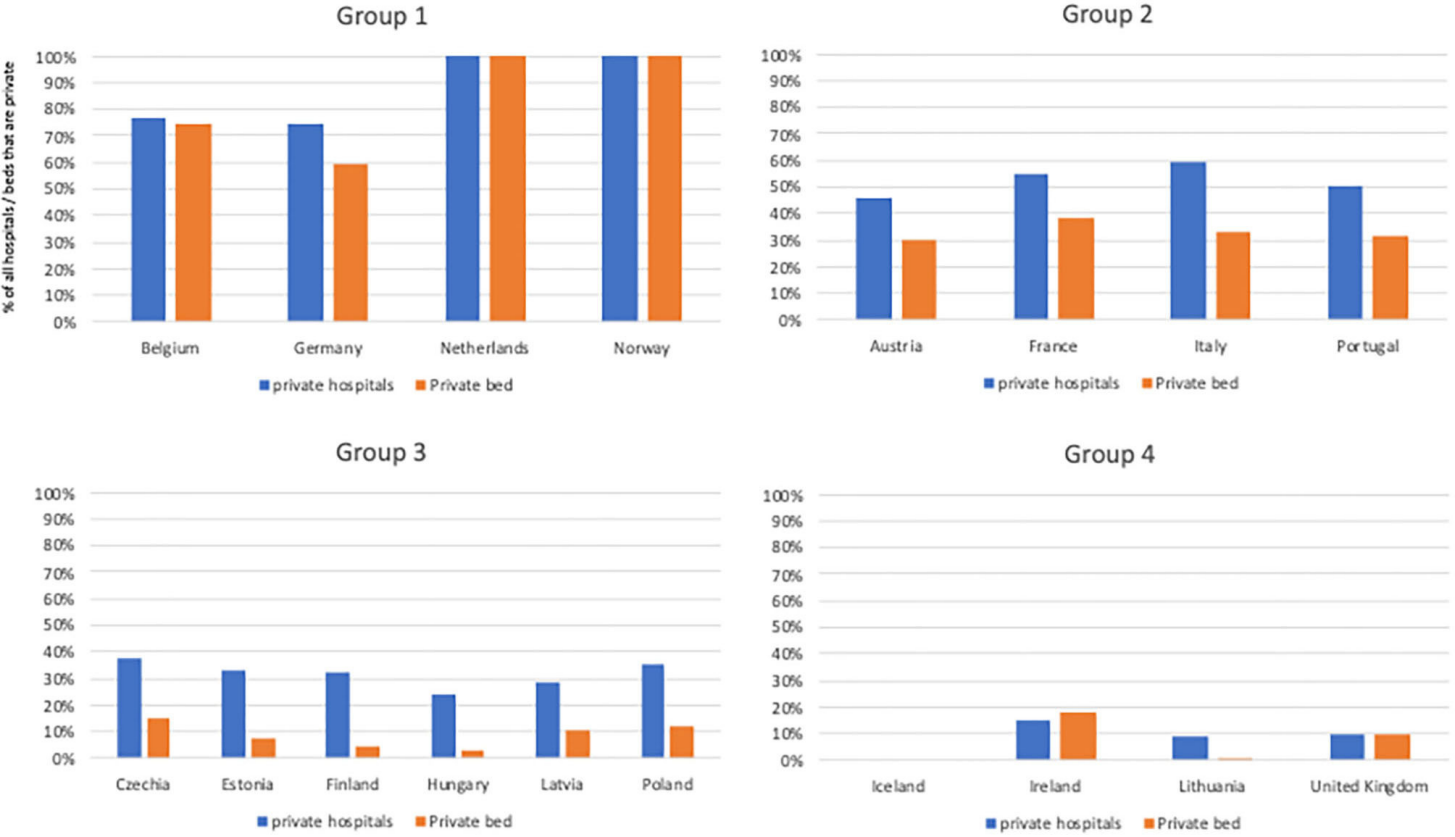
Percent of all hospitals that are private and percent of all hospital beds that are private. Where private beds are much smaller than hospitals, hospitals focus on outpatient care. Data not available for: Denmark, Greece, Luxembourg, Slovenia, Slovakia, Spain, Sweden, and Switzerland.

The behavior of private hospitals differs between the four groups, as can be seen in how private hospitals contribute to available inpatient bed within each group (Figure 3). In some countries private hospitals provide inpatient beds and services in proportion to their importance within the overall system; in other countries private hospitals have very few beds, focusing instead on outpatient care only. In Group 1, the private hospitals beds roughly match the private hospital numbers: this is where most inpatient care of all kinds is offered. Where public and private hospitals exist in parallel, as in Germany, the differences in services offered, bed numbers, bed-stay duration, and patient experiences between public and private hospitals are minimal: to the consumer and the social health insurance payer, public and private facilities are functionally equivalent. These countries' health systems are based on Bismarck's model of care and financing.

In Group 2, this equivalence exists for some services, or in some regions, but is not universal. In these countries private facilities increasingly have taken on profit-making outpatient services, often surgeries that have few co-morbidities and predictable management but also including delivery services and (among non-profits) some cancer management. The result of this can be seen in the average facility size: private hospitals in Group 2 have fewer beds than government facilities, and higher bed turnover reflecting their emphasis on outpatient and clearly defined, lower risk, care (26). These countries' health systems are heavily influenced by the Bismarckian model.

In Group 3 this same leaning away from comprehensive inpatient services and toward a narrower set of short-stay areas of care continues. Facilities are smaller and more specialized; non-profits are less predominant within the overall mix of facilities. While private hospitals exist, they offer fewer inpatient stay opportunities and take on fewer inpatient, chronic, or emergency services. Health systems in Group 3 countries have, largely, derived from the Semashko model which influenced much of Eastern European social services during the 20th century (Box 1) (27).

Box 1 **Countries in Transition: Group 3 Hospitals (25).**

A decade ago, the countries in Group 3 would have been called “economies in transition” from planned economies, organized around government provision of social services, including health, to market economies. It may be, then, that Group 3 will shift, or has already shifted, in ways not reflected in our data from 4 or 5 years ago, toward or away from Group 2.

The Group 4 countries are all countries with a strong national emphasis on social services. This group also includes many of Europe's small and isolated countries. For these, centralized management of a limited number of facilities is practical and competition unlikely to be an effective complement to government purchasing. In all Group 4 countries private hospitals either don't exist at all (Iceland), or exist as a small minority of facilities, principally serving only private patients for services not covered by national insurance (eg: cosmetic surgery) or outpatient services for patients who are willing to pay to avoid the wait times for government care. Health systems based upon Beveridge.

The differing role of private hospitals can be seen in the different ratio of beds-per-hospital shown in Figure 3, using the most recent data from each country. Ireland appears to be an anomaly; the only country where the private sector has more beds/facility than the public, although as elsewhere these beds are primarily for short-term services (28, 29). The very low percentage of private beds in all Group 3 countries indicates that in all of these countries private hospitals exist, but largely to provide outpatient surgeries and consultations.

### Dentists

Nearly all dentists in Europe work privately either in solo or group practices. In France 91% of the country's dentists are self-employed private practitioners (26). In Czechia the rate is 95%, in Austria 80%. Other than a few within hospitals, nearly 100% of dentists are private practitioners in Iceland, Italy, Lithuania, Luxembourg, Netherlands, Portugal, Greece, Germany, Spain, and the UK (10, 11, 30–37). The exceptions are few. In Finland private practitioners represent just more than half of all dentists and provide approximately one half of all dental care (38, 39). While there is some concern within the dental profession regarding how the growth in third-party payments will affect practices, most dental services across Europe continue to be funded by a mix of direct patient payment and government subsidy (40). Dental services for children up to 18 are government funded in all European countries (41). In Italy and Greece, dental services are nominally free within the government sector, but long wait times leads many patients to seek care from private offices (41). In the UK, dental care has been included in National Health Service (NHS) funding since 1948, however as in other countries, since 1951 adults have a co-payment required for non-acute services (41).

### Specialist Services

Data on specialist services (Table 1) comes from the OECD health systems survey (8). It found that in more than half of surveyed European countries specialists operate in private practice, either as solo practitioners (9/22 countries) or in groups (3/22). The countries where government specialist services dominate are all either in Group 2 (Italy, Spain, Portugal), Group 3 (Czechia, Finland, Hungary, Poland), or Group 4 (Ireland, Sweden, UK).

**Table 1:** Principal mode of specialist and primary care provision.

	**Primary care**	**Specialist care**
**GROUP 1**		
Belgium	Private Solo Practice	Private Solo Practice
Germany	Private Solo Practice	Private Solo Practice
Netherlands	Private Group Practice	Private Group Practice
Norway	Private Solo Practice	Private Solo Practice
Switzerland	Private Solo Practice	Private Solo Practice
**GROUP 2**		
Austria	Private Solo Practice	Private Solo Practice
France	Private Solo Practice	Private Solo Practice
Italy	Public Center	Public Hospital
Luxembourg	Private Solo Practice	Private Solo Practice
Portugal	Public Center	Public Hospital
Spain	Public Center	Public centers
**GROUP 3**		
Czechia	Private Solo Practice	Public Hospital
Finland	Public Center	Public Hospital
Hungary	Private Solo Practice	Public Center
Poland	Public Center	Public Center
**GROUP 4**		
Denmark	Private Group Practice	Private Solo Practice
Iceland	Public Center	Private Group Practice
Ireland	Private Solo Practice	Public Hospital
Sweden	Public Center	Public Hospital
United Kingdom	Private Group Practice	Public Hospital

*Comparable data not available for: Estonia, Greece, Latvia, Lithuania, Slovenia, and Slovakia*.

### Primary Care

The 2008/9 OECD health systems survey found that primary care services were predominantly provided in private settings in 15 of the 22 European countries, including almost all countries with social health insurance systems and five countries with national health systems: Denmark, Ireland, Norway, France, and the United Kingdom. In Finland, Iceland, Italy, Poland, Portugal, Spain, and Sweden primary care is mostly public (Table 1).

In Sweden, primary care is provided by health centers, comprised of a multidisciplinary workforce including general practitioners, nurses, specialist nurses with expertise in diabetes or other chronic illnesses, and often occupational therapists and psychologists. In 2019, 56.2% of Sweden's 496 primary care centers are public. The remaining 43.8% are private, operating under contracts with a region (42).

### Pharmacy

Outside of hospitals, community pharmacies across Europe are all privately owned and operated. There remain country variations in ownership restrictions, with Spain, France, and other countries restricting ownership by corporate chains and franchise arrangements as a way to protect and encourage local ownership (43). Eighty-five percent of the 145,143 pharmacies in Europe are private. Of these private pharmacies, one in three are affiliated with a franchise or other shared brand and one in eight are part of a chain (44, 45). The retail pharmaceutical component of the health system is sometimes inefficient, inequitable, unevenly distributed, and expensive. But it mostly works, and despite some shortcomings pharmacies function much like groceries, bakeries, or other commodity retailers. As a result most countries in Europe regulate pharmacies as a traditional, privately owned, market (46). The case study of Estonia, which liberalized its pharmacy market between 1993 and 1995 after gaining independence from the USSR, showed private ownership resulted in greater use, lower cost to the consumer, and greater client satisfaction (47). However, by 2014 regulation was needed to correct for market failures. Specifically, rural communities unserved by pharmacies were able to apply to the State which then mandated pharmacy chains meet certain size criteria to open a pharmacy in those regions (48).

In Sweden, a similar transition occurred. Until 2009 all pharmacies were government owned as part of the National Corporation of Swedish Pharmacies. From 2009, half of the government pharmacies were sold, and new private pharmacies were permitted. The total number of pharmacies increased by 20% in the following year and by 2011 there were 13 pharmacy operators in the country (49). The trend toward greater free-market structuring of pharmacies, and adaptive regulation to correct for market failings, has occurred across most countries of Europe, albeit at differing rates.

### Satisfaction

Gallup Poll data from 2016 shows high levels of satisfaction with national health services in Group 1 countries with high levels of private hospitals, private primary care, and private specialist services; but equally high satisfaction numbers in some countries within Groups 2 and 4 (Table 2) (50). Past studies have concluded that what European patients value most is choice and low out-of-pocket costs, and these are determined more by financing policies than service ownership arrangements (51).

**Table 2:** Citizen satisfaction with the health care system, 2016.

**Group 1**	**AVG**	**89.4%**
Belgium		91%
Germany		88%
Netherlands		86%
Norway		89%
Switzerland		93%
**Group 2**	**AVG**	**73.7%**
Austria		88%
France		78%
Italy		56%
Luxembourg		86%
Portugal		63%
Spain		71%
**Group 3**	**AVG**	**60.5%**
Czechia		72%
Finland		77%
Hungary		50%
Poland		43%
**Group 4**	**AVG**	**73.2%**
Denmark		85%
Iceland		67%
Ireland		60%
Sweden		78%
United Kingdom		76%

*Source: Gallup World Poll, cited in OECD “Government at a Glance” (50) statlink: http://dx.doi.org/10.1787/888933533834*.

## Conclusions

The delivery of healthcare in Europe, from hospitals to primary care to specialty services to pharmacies, demonstrates that while there have been and remain significant variations in how the private sector is engaged to provide healthcare within the larger health system, the variety can be taken to show that there are many ways to effectively deliver care. The private sector is neither necessary for the provision of national health care, nor is private sector service an impediment to a strong and effective national healthcare system. That can be said about hospitals, where the distinctions between ownership models are most stark and most clearly determined by national policy differences and changes. It can also be said for the provision of primary and specialty care, where the degree of private provision has historic roots, but both public and private models appear to deliver effective equity, access, and care (20).

At the same time, there is a near-universal accord within European health systems that the provision of community pharmacy and dental services are best served by private markets. These services and products are the most standard between providers, and hence the easiest for both purchasers and citizens to compare based on cost and accessibility. Among all healthcare goods and services, these behave the most like traditional market-based products and economists argue that private provision is the most efficient delivery option for this reason, something the European experience appears to confirm (52).

Case studies are critical for the many LMIC countries current expanding national and social health insurance, increasing investments, and revising regulatory systems to advance toward Universal Health Coverage in alignment with the Sustainable Development Goals. The European examples provide a critical insight for these governments: large scale privately provided medical services are neither necessary for achieving UHC nor a barrier to it. For any country now pursuing UHC, historical experiences and path dependency may dictate whether the private sector is an important provider of care. This was the case across the countries studied here. The varied models, and success, of Europe show that any extant delivery mix can be managed. Well-planned national policies and financing can assure effective universal coverage regardless of any inherited delivery structure.

This study offers a foundation on which further analysis should be conducted. We hope future efforts will assess the applicability of the system categories developed for Europe to countries in Asia, Africa, and Latin America.

## Author Contributions

DM was solely responsible for the analysis and writing.

## Conflict of Interest

The author declares that the research was conducted in the absence of any commercial or financial relationships that could be construed as a potential conflict of interest.
